# Magnetic Field Sensors Based on Giant Magnetoresistance (GMR) Technology: Applications in Electrical Current Sensing

**DOI:** 10.3390/s91007919

**Published:** 2009-10-12

**Authors:** Candid Reig, María-Dolores Cubells-Beltran, Diego Ramírez Muñoz

**Affiliations:** Department of Electronic Engineering, Universitat de València, C. Dr. Moliner, 50, Burjassot, Spain; E-Mails: m.dolores.cubells@uv.es (M.-D.C.-B.); diego.ramirez@uv.es (D.R.)

**Keywords:** physical sensors, magnetic field sensors, giant magnetoresistance, current sensors

## Abstract

The 2007 Nobel Prize in Physics can be understood as a global recognition to the rapid development of the Giant Magnetoresistance (GMR), from both the physics and engineering points of view. Behind the utilization of GMR structures as read heads for massive storage magnetic hard disks, important applications as solid state magnetic sensors have emerged. Low cost, compatibility with standard CMOS technologies and high sensitivity are common advantages of these sensors. This way, they have been successfully applied in a lot different environments. In this work, we are trying to collect the Spanish contributions to the progress of the research related to the GMR based sensors covering, among other subjects, the applications, the sensor design, the modelling and the electronic interfaces, focusing on electrical current sensing applications.

## 1. Introduction

Nowadays, due to the requirements of the novel applications, traditional magnetic field sensing methods are being revised and often substituted by emerging technologies [1].

Focusing on solid state magnetic sensors, magnetodiodes, magnetotransistors, Hall effect devices and magnetoresistors must be considered. Among them, Hall effect sensors are well established in industry while magnetoresistive sensors are nowadays continuously gaining supporters. Being both technologies compatibles with current CMOS fabrication processes, magnetoresistive sensors offer some intrinsic advantages. At room temperature, magnetoresistive sensors are, generally, more sensitive than Hall effect based ones, so avoiding the need of major amplification. Moreover, higher scale of integration can be achieved with novel magnetoresistance devices. In addition, in-plane fields can be measured with magnetoresistive structures, which is an advantages in, for example, electrical current sensors [2].

### 1.1. Magnetoresistance

The basic principle of the magnetoresistance (MR) is the variation of the resistivity of a material or a structure as a function of an external magnetic field, as generally described by the following general equation:

(1) R=f(B)

This definition is including a lot of different mechanisms producing this macroscopic effect. Nevertheless, the magnetoimpedance, a phenomenon consisting of the change of the total impedance *Z = R* + *jX* (where *R* is a real and×is an imaginary components) of a ferromagnetic conductor in a magnetic field, *H_ext_*, when a high frequency alternating current I = *I*_0_*e^−iwt^* flows through it [3], should not be considered as magnetoresistance.

The magnetoresistance can be found in classical semiconductors and, particularly, in magnetic semiconductors [4]. As for the Hall effect, its origin is in the Lorentz force. The deviation of the current path due to the magnetic field produces an increase of the current path length and, then, an increase of the effective resistance, described by

(2) $$R=R_{0}\frac{\rho_{B}}{\rho_{0}}\left((1+C_{1}{\left(\mu B\right)}^{2}\right)$$

where *R*_0_ is the resistance at null field, *ρ_b_/ρ_0_* is the specific relative resistance and *C*_1_ is a geometrical parameter. The use of this principle in magnetic sensing is very limited by the low MR level.

### 1.2. Anisotropic Magnetoresistance (AMR)

The Anisotropic Magnetoresistance (AMR), a typical effect in ferromagnetic materials, was discovered in 1857 by William Thomson. The anisotropic term is from its dependence from the angle between the electrical current and the magnetization direction. The AMR effect is described as a change in the scattering due to the atomic orbitals, caused by a magnetic field. This way, the resistance is at maximum when both directions are parallel and is at minimum when both directions are perpendicular. Mathematically speaking:

(3) $$R=R_{0}+\Delta R\cos^{2}\theta$$

This function displays maximums at angles of 45^°^. To achieve this enhancement in the response, the devices should be arranged in a barber-pole configuration, in which the current is forced to flow in a direction that is 45^°^ tilted with respect to the magnetic field. The most of them use permalloy as sensing material, deposited onto Si substrates in a Wheatstone bridge configuration. Typical magnetoresistance levels are close to 1%. This linear response is good enough for allowing the use of AMR devices in practical applications.

### 1.3. Giant MagnetoResistance

In 1988, Baibich et al. [5] and Binasch et al. [6] reported for the first time on what they called ″Giant″ magnetoresistance measured on Fe/Cr thin multilayers. They demonstrated that the electric current in a magnetic multilayer consisting of a sequence of thin magnetic layers separated by equally thin non-magnetic metallic layers is strongly influenced by the relative orientation of the magnetizations of the magnetic layers (about 50% at 4.2 K). The cause of this giant variation of the resistance is attributed to the scattering of the electrons at the layers interfaces. This way, any structure with metal-magnetic interfaces is a candidate to display GMR. Since then, a huge effort has been carried out in finding structures to enhance this effect (MR levels at room temperature above 200% are achieved in modern GMR structures). In the following, some of these structures are described, focusing of those with potential application in magnetic field sensing in general and current monitoring in particular.

#### 1.3.1. Multilayer

**Figure 1.**
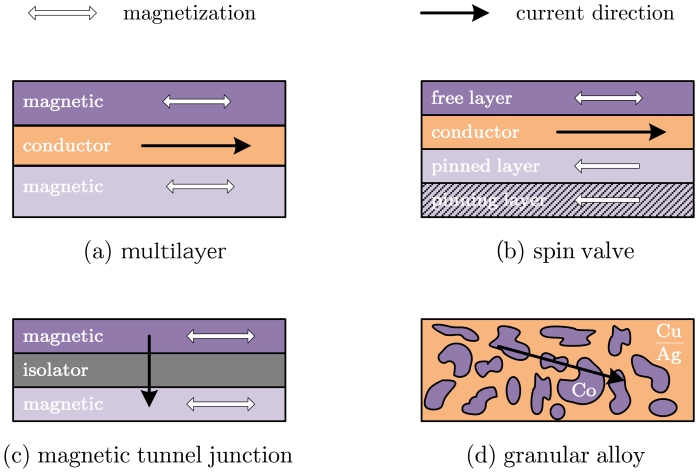
Basic GMR structures.

A multilayered structures consist of two or more magnetic layers of a Fe–Co–Ni alloy, as can be permalloy, separated by a very thin non magnetic conductive layer, as can be Cu [7]. A general scheme is shown in Figure 1(a). With magnetic films of about 4–6 nm width and a conductor layer of about 3–5 nm, magnetic coupling between layers is slightly small. With this configurations, MR levels of about 4%–9% are achieved, with linear ranges of about 50 Oe. The figures of merit of these devices can be improved by continuously repeating the basic structure.

Multilayered copper–permalloy (Ni_80_Fe_20_) [8] and copper–cobalt [9] structures have been developed by Mujika et al., following the early works of Piraux et al. [10] and Blondel et al. [11]. The films were deposited by DC sputtering onto thermally oxidized silicon wafers. The use of CMOS compatible substrates allowed the lithographic definition of meander-like magnetoresistances, deposition of platinum contacts and passivation with Si_3_N_4_. A final annealing step at 300 ^°^C for two hours in a controlled formingas (N_2_–H_2_) atmosphere conferred the samples a higher thermal stability and better magnetoresistance levels (1.1% at room temperature, B = 10 kG).

Successful applications of multilayered structures in magnetic field sensing include bio-electronics [8, 9] and angle detectors [12].

#### 1.3.2. Spin valve

The origin of spin valves are a particular case of multilayered structure [13]. In spin valves, an additional antiferromagnetic *(pinning)* layer is added to the *top* or *bottom* part of the structure, as shown in Figure 1(b). In this sort of structures, there is no need of an external excitation to get the antiparallel alignment. In spite of this, the pinned direction (easy axis) is usually fixed by raising the temperature above the knee temperature (at which the antiferromagnetic coupling disappears) and then cooling it within a fixing magnetic field. Obviously, so obtained devices have a temperature limitation below the knee temperature. Typical values displayed by spin valves are a MR of 4%-20% with saturation fields of 0.8-6 kA/m [14].

For linear applications, and without excitation, pinned (easy axis) and free layers are arranged in a crossed axis configuration (at 90^°^). The response this structure is given by [15]:

(4) $$\Delta R=\frac{1}{2}\left(\frac{\Delta R}{R}\right)R_{□}\frac{iW}{h}\cos(\Theta_{p}-\Theta_{f})$$

where *(ΔR/R)* is the maximum MR level (5%-20%), *Rn* is the sensor sheet resistance (15-20 Ω/n), *L* is the length of the element, *W* is its width, *h* is the thickness, *i* is the sensor current, and Θ*_p_* and Θ*f* are the angle of the magnetization angle of pinned and free layers, respectively. Assuming uniform magnetization for the free and pinned layers, for a linearized output, Θ*_p_* = π/2 and Θ*_f_* = 0.

As a practical example, in [16], the spin valve structure was deposited by ion beam sputtering (IBD) onto 3″ Si/SiO_2_ 1500 Å(1) substrates with a base pressure of 1.0×10^−8^−5.0×10^−8^ Torr. For IBD deposition, a Xe flow was used for a deposition pressure of 4.110 5Torr. The spin valve structure was Ta(20 A) / NiFe(30 Å) / CoFe(20 Å) / Cu(22 Å) / CoFe(25 Å) / MnIr(60 Å) / Ta(40 Å). This structure has demonstrated to give magnetoresistance responses of about 6%−7%, linear ranges of about 20 Oe and sheet resistivities of about (10-15 Ω/□) [16]. Deposition rates ranged from 0.3 Å/s to 0.6 Å/s. A 40 Oe field was applied to the substrates during the deposition step in order to state the easy axis in the pinned and free layers. The wafer was 90 ^°^ rotated between both depositions to ensure a crossed-axis spin valve configuration.

Nano-oxide layers (NOL) inserted in the pinned layer and above the free layer have been found to increase the magnetoresistance ratio [17]. The enhancement of GMR is attributed to the specular scattering effect of the conduction electrons at the metal/insulator interfaces.

In [2], the specular spin valve structure was Ta(3 nm) / NiFe(3 nm) / MnIr(6 nm) / CoFe(1.6 nm) // NOL // CoFe(2.5 nm) / Cu(2.5 nm) / CoFe(1.5 nm) / NiFe(2.5 nm) // NOL // CoFe(2.0 nm) / Ta(0.5 nm). NOL layers were formed in a 15 minutes natural oxidation step at atmospheric pressure in the deposition tool load lock. The natural oxidation process, keeping its simplicity, has proven to be well effective. Finally, the samples were annealed at 270 ^°^C under vacuum and cooled under a 3 kOe magnetic field applied parallel to the pinned and free layer easy axis.

#### 1.3.3. Magnetic tunnel junctions

This case, the magnetic layers are separated not by a conductive layer but a very thin isolating one, following a CPP configuration (See Figure 1(c)). Electrons can surpass this thin film by means of the quantum tunnel effect [18]. As deducted from quantum mechanics arguments, the crossing probability is higher when both magnetic moments are aligned in parallel and lower when both magnetic moments are not aligned in parallel. This devices usually make use of the spin-valve principle in order to fix the easy axis by means of a pinning antiferromagnetic layer. Typical MR levels of MTJ are above 40%, with Al_2_O_3_ as isolating layer [19]. More recently, MR levels about 200% have been reported for MgO based structures [20]. Saturation fields are in the order of 1-100 Oe.

The basis of linear magnetic tunnel junctions is analogous to that of linear spin valve. When configured in a crossed axis configuration, linear ranges suitable for sensor applications can be achieved [15]. Nevertheless, the usage of linear MTJ is still in its initial stage and is demanding additional research efforts.

In [21], the MTJ structure was deposited by ion beam sputtering (IBD) onto 3″ Si/SiO_2_ 1000 Å substrates. The final structure of the MTJ was: Al (600 Å) / Ta (90 Å) / NiFe (70 Å) / MnIr (250 Å) / CoFe (50 Å) / Al_2_O_3_ (12 Å) / CoFe (50 Å) / NiFe (25 Å) / Ta (60 Å) / TiW (300 Å). This structure has demonstrated to give magnetoresistance responses close to 40% whereas keeping linear ranges above 20 Oe and RAP of several hundreds of *μ*m^2^.

#### 1.3.4. Granular alloys

Granular films of Co–Cu and Co–Ag also exhibit a giant magnetoresistance effect [22]. In this case, the giant magnetoresistance effect is due to the spin-dependent scattering taking place at the boundaries of Co clusters embedded in the host lattice, as depicted in Figure 1(d). Because of these binary systems are not miscible, the characteristics of the devices are highly conditioned by the growth conditions and the post-deposition treatments. In fact, the amount of magnetoresistance is accepted to be associated to the size of the Co clusters [23].

Vergara *et al.* obtained granular films of Ag–Co and Cu–Co (10% Co) by pulsed laser ablated-deposition [24]. A pulsed Nd–YAG laser was used. The considered substrate was common glass. The process was carried out at room temperature (10–5 mbar), with deposition ratios of the different elements ranging from 0.04 nm/s to 0.08 nm/s. So obtained films (from 50 to 75 nm thick) needed a post-annealing process (10 min at 500 ^°^C in Ar atmosphere) before their characterization. Scanning tunneling microscopy revealed the existence of 30 nm diameter and 10 nm high grains. Magnetoresistance levels up to 9% at 77 K in a 10 kOe magnetic field were reported.

Andrés *et al.* used RF sputtering for obtaining Co–Cu (25% Co) granular films onto unheated glass substrates. Deposition ratios ranged from 0.15 nm/s to 0.75 nm/s (4 × 10^-7^ Torr). This case, the cluster radius were about 20 Å, and magnetoresistance values up to 9% at room temperature in a 15 kOe magnetic field were measured.

Arana *et al.* deposited Co–Cu (30% Co) granular films onto a polished alumina (Al_2_O_3_) substrate. This deposition was demonstrated to be compatible with some subsequent microfabrication processes (lithography, contact deposition and passivation) in order to obtain functional sensors [25, 26]. The same research group also studied the influence of the deposition temperatures on the performance of the films [9].

#### 1.3.5. Other exquisite structures

Giant magnetoresistance is also found in other structures. We collect three illustrative examples. Pena *et al.* [27] report on giant magnetoresistance in ferromagnet/superconductor superlattices. On the other hand, Pullini et al. [28] describe GMR in multilayered nanowires. Thirdly, Svalov et al. report on successful spin-valve structures with Co–Tb based multilayesr [29]. In any case, a magnetic/ non-magnetic interface is required in order to allow the spin-electron scattering producing the effect.

## 2. Sensor Design

The design and development of a GMR based sensor depends on considerations coming from different involved fields in order to get functional devices. Moreover, the specific design will be necessarily linked to the particular application for which it is designed. Every part of the sensor design will have bigger or lesser repercussions on the final device performance. For example, both the linear range and the thermal characteristics of the sensors will be functions of the characteristics of the sensing structure as well as the final encapsulation. A more detailed knowledge of these parameters is absolutely necessary prior to the starting of the sensor development.

### 2.1. Bridge configuration

Even though a unique resistance can be used as sensing element, a Wheatstone bridge setup is always a good recommendation as the starting step in the design of resistive sensors. This case, we will get a differential output as a function of the resistance variation. Depending on the considered case or the particular requirements, we can make use of several bridge configurations. Table 1 displays a summary of possible bridge configurations with calculated output voltage. As easily observed, a full bridge configuration is the best choice in terms of signal level and linearity (see Table 1, right). Nevertheless, and in the case of GMR, often it is impossible to take advantage of such setup. Because of the orientation dependence in the fabrication process of GMR structures, a half bridge configuration with two active resistances and two shielded ones is used (see Table 1, center) [30]. If a two steps deposition is assumed, a full bridge can be obtained.

### 2.2. Device arrangement

As the range of applications increase, the sensor development process becomes more and more specific. As can be easily understood, a bioelectronics application requires a particular treatment that should be different than, for example, an electrical current sensing application.

In any case, a GMR sensor is a magnetic field sensor. These sensors can be used for detecting a magnetic field or a disturbance in the earth magnetic field produced by a magnetic issue. This way, the design of the sensing system is, in the most of the cases, ad hoc.

#### 2.2.1. Open design

The first approach for implementing a GMR sensing device is to use a common (commercial) magnetic field sensor. We can also find Hall based electrical current sensors sharing this philosophy.

In [31], two AB001-02 GMR gradient sensors from NVE [30] are used in the design of a traffic speed monitoring system. The sensors are placed in a standard PCB with the necessary electronics. In this case, the separation between both sensors and the orientation of the sensors for the highest sensitivity are the key points in the design. On the other hand, for implementing a vibration detector based on GMR sensors, commercially available GMR magnetic sensors (SS501, from HuaXiaMag) were used in [32]. Three different sensors were geometrically arranged in order to cover the three spatial directions.

For specific electric current sensing, soldering the magnetic sensor onto a simple current PCB strip is the common way to implement a measuring system. This straightforward approach is followed in [33], where a AC004-01 from NVE is used for managing the charge and discharge process of a battery. A similar scheme is used in [34] for the conductance control of switching regulators. Being a simple way to construct a sensor, this kind of configuration displays some disadvantages. The first one is that the system is very sensitive to fabrication tolerances. A calibration procedure, not always easy to carry out, needs to be done after the installation. In addition, open designed electrical current sensors are very sensitive to external interferences. Because a typical magnetic sensor is the basic sensing element, any external magnetic field will be added to the current signal and disturb the measurement. When the external magnetic sources are known, they can be eliminated by geometrical arrangements, taking advantage of the directionality of the sensors. If, like in the most of the cases, this sources are unknown, other solutions must be considered. An additional magnetic probe can be placed close to the sensor, but out of the scope of the magnetic field generated by the current. We can also use to identical sensors placed oppositely onto the current track and then to perform a differential measurement. A good review of this sort of approaches can be found in [35].

#### 2.2.2. Partially compact

As an intermediate approach, and if we have access to the sensor microfabrication process, we can consider the design as a mixed microelectronic/mechanical design procedure.

Bio-chip applications need to be specifically considered. Microfluidic devices require a multilayer technology compatible with CMOS-MEMS structures in order to achieve the integration of different functions and materials in a Lab On Chip. The sensing elements need to be placed close to the container containing the fluid to be analysed. The use of microchannels, magnetic markers and auxiliary external magnets are commonly used in this sort of devices. The appearance of epoxy-resin photoresists used jointly with polyimide films have allowed the fabrication of high-aspect-ratio microstructures by standard UV lithography [8, 9].

**Figure 2.**
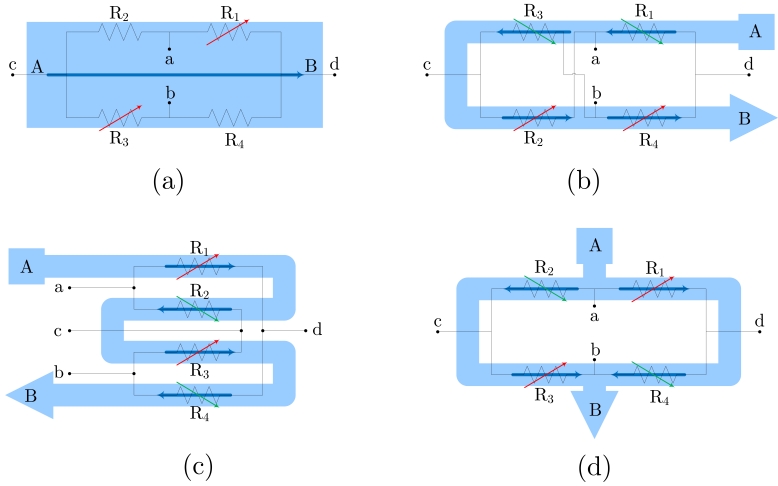
Different current sensor configurations as a function of the current path arrangement (*a, b, c* and *d* are the bridge contacts; *a* and *B* are the current path ends). (a) Straight path (half-bridge behavior), (b) 'U' shaped path (bridge contacts must be rearranged), (c) 'S' or serial shaped path, (d) parallel shaped path.

Regarding electric current sensors, if the current paths are considered as a sensor design step, we can include it in the encapsulation process. This way, a better control can be applied to the overall fabrication process and lower manufacturing tolerances are expected. So the obtained sensors then include two new terminals (input and output) for the current. The simplest way to do it is shown in Figure 2(a), assuming a half bridge operation. By geometrical considerations, a full bridge response can be obtained, as detailed in Figure 2(b)–(d).

#### 2.2.3. Fully integrated

**Figure 3.**
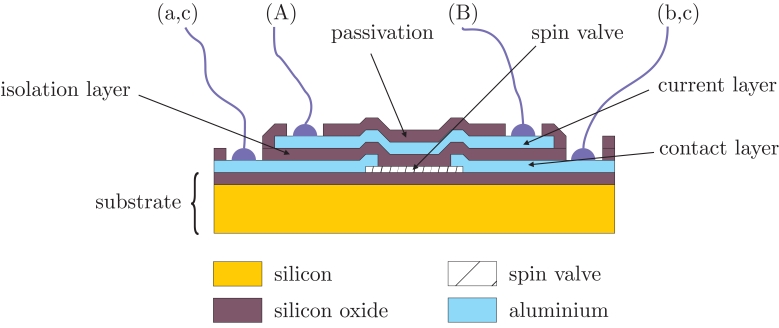
Cross section a of a current sensor with integrated current straps. Nomenclature detailed in Figure 2.

When specifically dealing with electrical current sensors, we can directly incorporate the current strips into the integrated circuit during the microfabrication process. As an illustrative example, the cross section of a GMR low current sensor, as reported in [2], is shown in Figure 3. Even though the process is described for a spin-valve based device, it can be directly translated to MTJ or any other GMR structure. In addition, any of the geometrical arrangements shown in Figure 2 can be reproduced here.

The design of this sort of sensors is pretty delicate. The particular encapsulation of the device is on the origin the a number of undesired effects as can be: poor isolation due to an incorrect dielectric selection, thermal limitations originated by Joule heating, mutual couplings as a result of the closeness of the current paths, … Each effect should be separately considered and analyzed in order to obtain an optimal design.

## 3. Applications

The life of GMR based sensors is very short. In fact, the first commercial GMR sensors were introduced in 1995 [36]. The rapid evolution of GMR sensors technology has opened a wide and promising range of applications. Apart from electrical measurement related systems, GMR based sensors are nowadays being utilized in different fields as engineering, physics, biology, space, … The following sections are devoted to the direct and indirect contributions to Spanish research group in the field of GMR based applications.

### 3.1. Electrical current sensing

Traditionally, electrical current has been measured by means of shunt resistances, coils and solid state sensors. The Ohm's law is the basis of the first method and variations of the Faraday's law are applied to the second case. We will focus on the third option, where the magnetic field generated by a current flow is detected by a solid state magnetic sensor. This general scheme can be applied to the measurement of a current driven by a wire (Table 2, left) or by a conductive strap in a printed circuit board or an integrated circuit (Table 2, right). AC/DC currents can be measured in this way with small, cheap and contact-less systems. When dealing with GMR or GMI sensors, excellent sensitivities are achieved. For example, in [37], a current sensor based on the GMI characteristics of ferromagnetic wires is developed. When excited at 100 kHz, sensitivities of about 2.5 V/(V·A) in a 0–0.3 A range are demonstrated.

#### 3.1.1. Industrial electronics applications (large to medium currents)

As continuously suggested in this paper, the more straightforward application of GMR based electrical current sensors is the final implementation of an ammeter.

In [38], a specific spin valve sensor for industrial applications is designed, characterized, implemented and tested. The sensor was soldered onto a PCB current track and encapsulated chip-on-board, following the partially compact scheme previously presented. A full bridge (active in pairs) with crossed axis configuration was utilized. The sensor displayed a linear range up to 10 A.

In [16], a novel design principle is presented. The principle of operation is depicted in Figure 2(c). With this configuration, the current flows from-left-to-right above *R*_1_ and *R*_3_, and from-right-to-left above *R*_2_ and *R*_4_. Consequently, when a current is driven (from A to B), and depending on the sign, resistances *R*_1_ and *R*_3_ increase/decrease their values and resistances *R*_2_ and *R*_4_ decrease/increase their values, thus obtaining a full Wheatstone behavior. The sensor is fed through terminals *a* and *b*, and the output is taken between terminals *c* and *d*. Due to the particular arrangement of the magnetoresistors, this sensor is theoretically insensitive to external magnetic field, therefore minimizing the possibility of interferences (below 1% [16]). This particular approach has been successfully applied to PCB-IC mixed technology based moderate current sensors, with sensitivities close to 1 mV/(V·A).

##### Differential currents

Differential currents can also been measured with the help of GMR sensors [39]. A GMR sensor (AC004-01, from NVE) is placed into two Helmholtz coils, carrying the currents to be compared. When both currents are identical, the magnetic field in the middle point of the coils is zero, and so the output voltage of the sensor. The system was tested in a house-hold application, demonstrating to be useful for detecting differential currents below 30 mA.

##### Switching regulators

In [40] the performance of the sensor presented in [38] was compared with a common Hall effect current transducer (LEM, LA 55-PS/P1) within a high-frequency bi-directional three-phase rectifier, to be used in accelerator applications at the European Laboratory for Particle Physics (CERN) [40]. The spin valve sensor displayed excellent figures regarding noisy and heat environment, due to their intrinsic properties.

##### Wattmeters

The need for measurement of the active electric power led to the development of various types of power meters. The basic, classical electromechanical gauges using electrodynamic measurement system are still used, in spite of their drawbacks. The dynamic response of them is limited, since they employ coils of considerable inductance. Recently, the bandwidth of power meters became a crucial parameter due to the need of the measurement of general, rather non-harmonic signals with a high content of the higher harmonic frequencies. By that time, two basic approaches arose:

##### Independent voltage and current measurement

Separating real-time sampling and A/D conversion of the current *i (t)* and voltage *v (t)*, followed by fully digital processing of acquired data is the more straightforward scheme for power measurement. Is the approach followed, for example, by Ramírez *et al.* In [41], an electronic system to measure active, apparent and reactive energies and power delivered to an AC line load is presented. To measure the current, a signal conditioning circuit based on a magnetoresistance sensor (Zetex, ZMC20) is shown. A specific IC (ADE7753 from Analog Devices) is used for monitoring the energy. The system was successfully tested in the range of 220 V_RMS_ and 5A_RMS_.

##### Voltage and current measurement multiplying

**Figure 4.**
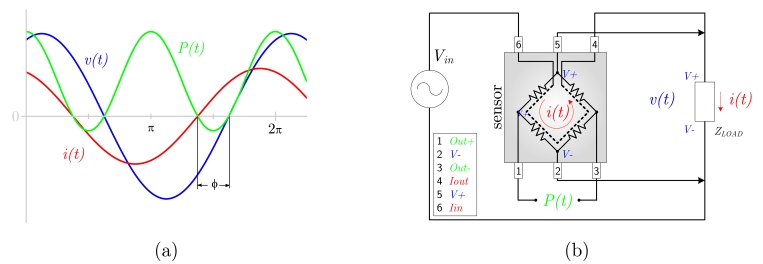
Power measurement with a MR sensor. (a) Description of instantaneous power, (b) Possible configuration with a Wheatstone bridge MR sensor.

By using of an appropriate analog electronic multiplier, two signals proportional to the voltage and current, respectively, can be multiplied in real-time, as suggested in Figure 4(a). Thus, the output of such a transducer is the instantaneous power of the signal defined as:

(5) p(t)=i(t)⋅υ(t)

Even though power transducers based on Hall sensors as multiplying elements can be used for direct power measuremtent, their insufficient sensitivity usually results in the need of ferromagnetic cores to concentrate the magnetic flux into the sensor area. The higher sensitivity of GMR based sensors makes them as potential substitutes of Hall sensors for this application.

The basic idea o_-_ using an MR element as an analog multiplier is very simple: the Wheatstone bridge of the MR sensor is supplied by a signal, which is proportional to the voltage of the measured signal. At the same time, current proportional to the current of the measured signal is led through a coil, generating magnetic field which is the Wheatstone bridge exposed to. The output (diagonal) voltage of the bridge is (linearly) dependent on the acting magnetic field, and at the same time, it is linearly dependent on the supplying voltage. As a direct consequence of these two facts, the output is dependent on the multiple of the two signals. A graphical scheme is presented in Figure 4(b). The idea has been recently applied by using a KMZ51 AMR based commercial sensor [42]. The substitution of the AMR by a GMR based sensor is currently under study.

An interesting application of GMR sensors for measuring and controlling electrical power during charging and discharging batteries is presented in [33]. A specific circuit is implemented for taking advantage of the multiplying characteristics of a GMR sensor. The system demonstrated its validity in 12 V batteries with chargin/discharging currents up to 4 A.

#### 3.1.2. IC current monitoring

SV based sensors have been successfully applied to low current measurement, in different scenarios [43], in particular some compatible with CMOS technology. As examples, we can consider the inclusion of these milli-Ammeters in GMR based systems-on-chip (SoC) for biological applications or the need of built-in current sensors (BICS) for built-in self testing of different families of integrated circuits (IC). Some work has been previously reported regarding the application of GMR based sensors to the electrical current measurement at the IC level. In fact, We recently demonstrated the applicability of spin-valve structures to the measurement of low electric currents [2, 44]. In these works, we introduced the concept and some fabrication parameters were established. In [45], the potentiality of SV based full Wheatstone bridges for low current monitoring at the IC level is demonstrated. A number of prototypes are specifically designed, fabricated and tested. The current lines are incorporated in the chip during the microfabrication process which reduces the separation to the sensing elements, leading to improved sensitivity. Therefore, in order to get a balanced bridge, the current paths need to be properly designed. The characteristics and geometry of these current paths have been considered as basic design parameters. Current ranges from 10 *μ*A to 100 mA can be covered with these sensors with excellent linearity and sensitivities above 1 mV/(VmA). AC characteristics have also been analyzed and bandwidths exceeding 100 kHz are demonstrated. In order to highlight the design properties, dependence of the sensor's performance with external magnetic perturbations and self-heating have also been measured and quantified. The associated errors are in the range of 1%–2% of the full scale.

##### Miliwattmeters

Following the technological scheme exposed in a previous paragraph, we can translate the multiplier characteristic of GMR devices introduced previously in order to implement micro wattmeters suitable to be integrated jointly with the CMOS circuitry. As the power source (AC or DC), any active portion of the IC can be considered. As the load, any fed portion of the IC can be considered (R-L-C).

The mathematical analysis of the circuit can be easily made with the previous equations. A non-galvanically isolated approach needs to be considered. This way, there is no need for an additional transformer and the functionality extends from DC to the frequency limit of the device. By contrast, a series resistance need to be added in order to match impedances and to limit the sensor power consumption. A detailed analysis of the different resistive losses must be done in order to fix the operation range of the system.

##### Electrical isolators

Signal isolator devices are widely used in many electronics systems. The more commonly used isolators are optical isolators (optocouplers) and capacitive or inductive couplers (purely transformers). Some common disadvantages of these devices are that they are often limited to linearity and frequency performance, they need a notable power consumption and they display a considerable size and usually require hybrid packaging, a real handicap for integrated circuit fabrication. In order to overcome these difficulties, the possibility of using magnetic tunnel junctions (MTJ) based full bridges in order to design analog magnetically coupled isolators is analyzed in [21]. MTJ display some advantages when compared with spin-valve, namely smaller devices and higher magnetoresistance levels (and then higher sensitivity devices). Nevertheless, the MTJ structures must to be carefully designed in order to obtain useful linear ranges. To preliminary demonstrate the capability of these devices for acting as analog isolators, different electrical signals were applied through the input terminals of specifically MTJ full bridge designed compact prototypes, with the help of a signal generator. A useful range up to 50 mA and 100 kHz was demonstrated.

### 3.2. Other than Electrical Current Sensing

#### 3.2.1. Civil engineering applications

The most of the applications developed with GMR magnetic field sensing is related to the measurement of the Earth's magnetic field perturbations produced by specifically considered ferreous body. This way, a position detecting scheme is always present.

For example, it is possible to use GMR sensors to locally measure the small magnetic perturbations caused by the iron of the car's body over the Earth's magnetic field. Moreover, if we use GMR gradient type sensors, the output signal is only dependent on the magnitude of the magnetic field variation, and no additional external magnetic field compensation is required. This way, a voltage ″signature″ is obtained from the differential output of such a sensor when a car is running close to it. Within this scheme, it is easy to incorporate another sensor, placed to a well known distance in order to also measure the car speed. This proposal has been successfully developed by Pelegrí *et al.* [31].

The same physical principle can be directly translated to the measurement of vibrations in industrial machines. The small magnetic variations over the Earth's field produced by the vibration of the ferromagnetic pieces in industrial installations can be converted into resistance variations by the use of GMR magnetic field gradient devices. By using three sensors with the appropriated XYZ arrangement, a complete description of the vibration can be obtained. A prototype was developed by Pelegrí *et al.* [32] and successfully tested with a drilling machine.

For linear magnetic position, in addition to the measurement of the Earth's field variations produced by magnetic materials, we can also use, if possible, permanent magnets associated to the moving part of the system. This way, the measurement of the absolute magnetic field is considered. Arana et al. [26] reported on the design of a high sensitivity linear position sensor using granular GMR devices. Sensitivities above 10 mV/V/mm are demonstrated by the utilization of Nd-Fe-B (0.4 T) magnets.

Angle and circular position detectors are also demanded by the industry: automotive applications, rotational machinery, … This kind of sensors are usually designed as contact-less systems in which a magnetic sensor (GMR in our case) detects the relative angular position of a rotationally moving magnet. This is the case presented in [25] and [12]. In the first case the authors focus on their specifically designed sensor, based on a granular MR. Because of the independence on the magnetic field direction, this technology is optimal for cylindrical symmetry problems. When a NdFeB is used, sensitivities about 0.25 mV/V/ ^°^ are achieved.

#### 3.2.2. Biological applications

With the rapid development of microfabrication techniques, together with the finding of compatible devices, the concept of Lab-on-a-Chip has become more and more important in the last years. Portable devices have been recently developed which are capable of driving a fluid through microchannels close to a detecting region, with additional conditioning and acquiring electronics. The usual scheme is the detection of the magnetic fringe field of a magnetically labeled biomolecule interacting with a complementary biomolecule bound to a magnetic field sensor. In this context, magnetoelectronics has emerged as a promising new platform technology for biosensor and biochip development [46].

Mujika et al. [8, 9] report on the detection of *Escherichia coli* O157:H7, an infectious agent that is present in several cook categories, such as meat and milk. Even though no field tests are reported, the developed system is fully described and characterized.

#### 3.2.3. Space applications

The conservative aerospace sector traditionally used old and well experimented components in its developments. The utilization of brand new technologies in commercial of the shelf (COTS) for space missions is nowadays only in the early stage. COTS are cheaper, faster in delivering and with wider reliability. Michelena *et al.* [47–49] introduce the possibility of using GMR commercial sensors in space applications. GMR sensors have not been flown yet but INTA, the Spanish National Institute of Aerospace Technology is working on the adaptation of a miniaturized GMR three axis sensor (HMC2003, from Honeywell) to the attitude control system in the frame of the OPTOS project, which is a 10 × 10 × 10 cm^3^ Picosat devoted to be technological test bed. The circuitry consist of conditioning and biasing electronics blocks.

## 4. Conditioning Circuits

Active research is also carried out regarding the electronics devoted to biasing and conditioning GMR based sensors. In the next paragraphs, some important results are described in terms of electrical biasing, basic conditioning (amplification and acquisition), linearizing and thermal compensation.

### 4.1. Biasing

Intrinsic characteristics of GMR sensors bring to the need of biasing techniques. Both an external magnetic field or an appropriate circuit can set the correct bias point of the sensor. In the first case we can use an external permanent magnet or an integrated coil in order to slightly displace the quiescent operation point of a sensor.

On the other hand, and assuming a resistor bridge configuration, a constant voltage source can be used to feed the sensor, through two opposite vertex of the bridge. The differential output voltage is taken from the remaining pins. Nevertheless, it has been demonstrated that thermal characteristics (temperature drifts) of spin valve based sensors are notably improved by using a constant current source for the sensor feeding [30].

**Figure 6.**
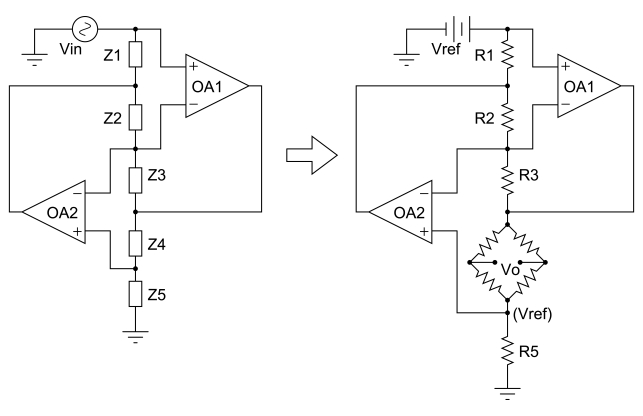
Generalized impedance converter (GIC) as a constant current biasing of magnetoresistive sensors (see the main text for details).

In this sense, Ramírez *et al.* have demonstrated the use of a classical Generalized Impedance Converter (GIC) Figure 6 (left) as a constant current drive for resistive sensors, by substituting any general impedance (*Z*_1_ to *Z*_5_) in the original configuration by resistances [50]. If we observe Figure 6 (right), and by considering a DC regime and ideal op amps, *V*_ref_ appears at all the op amp inputs, and then, it is transferred by op amp action from the GIC input terminal to resistance *R*_5_. Once *V*_ref_ and *R*_5_ are selected, current *I*_0_ through *R*_5_ is well defined. Then, all the current through *R*_5_ also circulates through *R*_4_ and, accordingly:

(6) $$I_{R_{4}}=I_{R_{5}}=I_{0}=\frac{V_{\text{ref}}}{R_{5}}$$

### 4.2. Conditioning: amplification and acquisition

When dealing with resistive bridges, for getting the differential output voltage, a differential amplifier is commonly considered. This solution is assumed, for example, in [31–33, 39, 50]. In some cases, the use of a differential band pass filter is also required. In the most of the cases, mainly when dealing with commercial sensors, a standard PCB technology is applied. For specific applications, an Application Specific Integrated Circuit can be developed [12].

### 4.3. Linearizing

As presented in a prior paragraph, GMR sensors are often developed as half- or full-bridge sensors in order to improve the linearity. As above commented, a DC (V or I) biasing is generally used as exciting source. Nevertheless, Sifuentes *et al.* [51] have recently demonstrated that by directly connecting a resistance to a microcontroller, the linearity can be improved by measuring the charging/discharging of a RC system.

### 4.4. Thermal compensation

Assuming that thermal effects cannot be completely eliminated, various methods of temperature compensation have been reported to reduce the thermal drift output of Wheatstone bridge type sensors. These methods can be classified as noninvasive or invasive. By noninvasive we mean technique consisting of the addition of different circuit elements in series or parallel to the bridge in order to reduce its thermal drift, as described, for example in [52]. A temperature sensor, a fixed resistor, some kind of active network (diode or transistor) or a fixed current source have been successfully applied. This way, the addition of one of the above elements results in a change of the bridge supply voltage due to the temperature variation, which produces a valid compensation. A slightly different approach consists of the connection of a temperature variable gain instrumentation amplifier in cascade at the output of the bridge. On the other hand, a Wheatstone bridge can also be temperature compensated by means of the modification of its original configuration. In this case, we should ensure that the terminals of the bridge are externally accessible. This group of techniques can be considered as invasive, because the conditioning circuitry in common commercial sensors make the bridge terminals often inaccessible. An excellent revision of these works is made in [53], where a novel application of the Generalized Impedance Converter (GIC) as a thermal compensating biasing circuit for specific magnetoresistive sensors is also presented. The method employs a temperature sensor external to the bridge and both components are placed inside the GIC topology. The resulting circuit is used by the bridge as a temperature dependent current source reducing its output temperature coefficient. The temperature coefficient of the sensitivity is hence reduced in a factor of three [53].

## 5. Simulation

Numerical modelling and simulation are useful tools than can be used both for design and analysis of GMR magnetic field sensors. These sensors can be analyzed from the physical or the electrical point of view.

### 5.1. Electrical modelling

**Figure 7.**
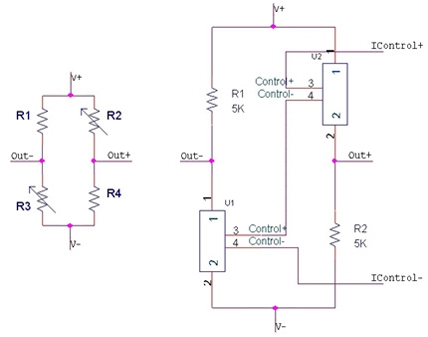

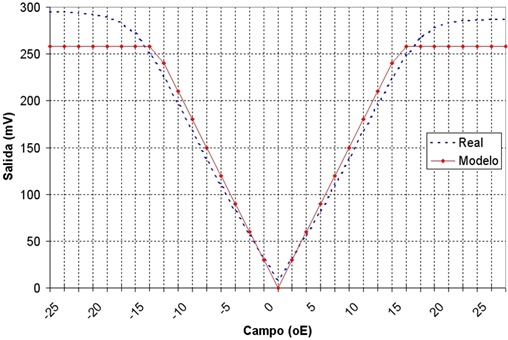

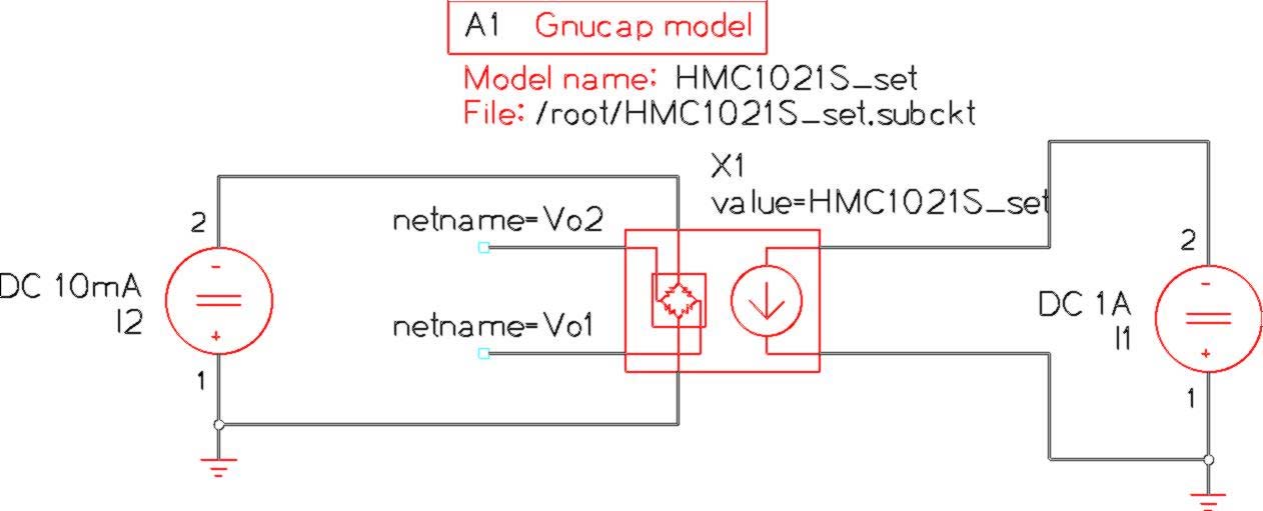

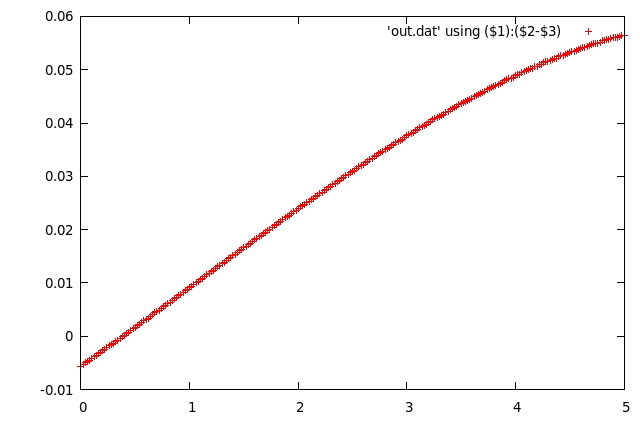
SPICE model of a NVE AA002 sensor and Gnucap model of a Honeywell HMC1021S sensor. Comparison with measured response.

One of the current aspects of electrical current sensors is the lack of digital models to be included in circuitry simulations (mainly SPICE) as developed for Hall based sensors. The underlaying physics of these devices make the task very difficult and only analog linear models have been created until now. Only specific current sensors are candidates to be modelled. For SPICE, resistances are native devices, so they cannot be reformulated without modifying the source code [54]. This research line remains open and further works should be required. By contrast, a linearized model of the GMR response can be introduced in a SPICE analog behavior models (ABM). When correctly defined, these ABM's works as black boxes performing the linear function for which have been programmed. This blocks can be as complex as necessary. In Figure 7 a basic SPICE model of a NVE AA002, used as a current sensor, is shown. The defining parameters have been experimentally determined and included in a simple linear model [35]. As a comparison, a HSPICE model for giant magnetoresistance memory bits was presented in [55].

*Gnucap* is an open source general purpose mixed analog and digital circuit simulator released under the GNU license. It is mostly SPICE compatible and also has a behavioral modelling language [56]. Among its capabilities, *Gnucap* include a voltage controlled resistor. Its behavior can be described by an expression or a table, which allows the modelling of GMR-based devices by including the input resistor in the model. Some research is being carried out in this sense by the authors. In Figure 7, some preliminary results are presented.

Recently, *Verilog-A* has emerged as a powerful hardware description language, suitable for analog applications. We are currently working in the development of a circuital model for this kind of current sensors.

### 5.2. Physical modelling

Among the many methods that can be applied to the modelling of this kind of sensors, the Finite Element Method (FEM) is usually preferred because of its flexibility and the good number of commercial software packages that are available. More specifically, the FEM has recently been applied to the analysis of different current sensors configurations [57].

**Figure 8.**
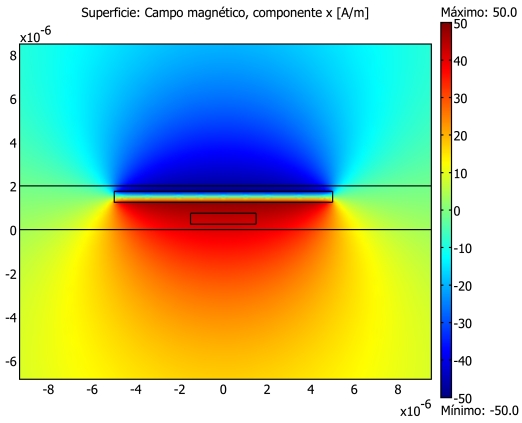

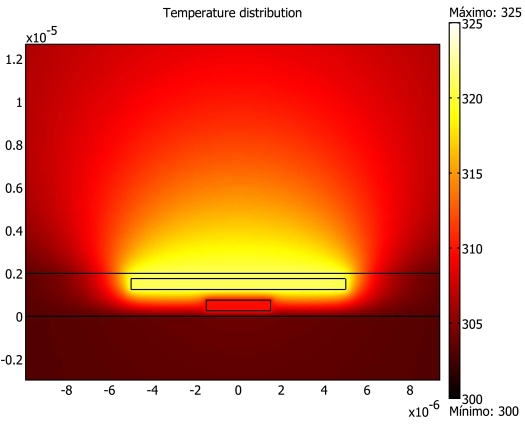

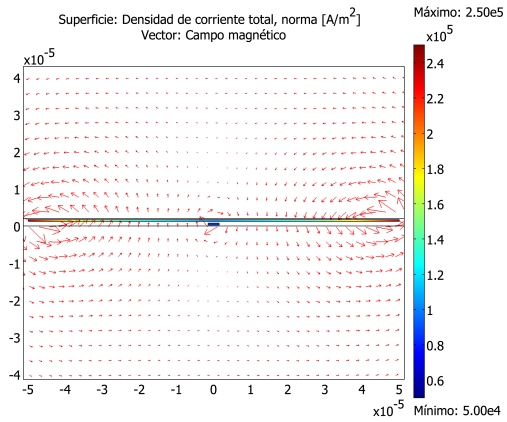

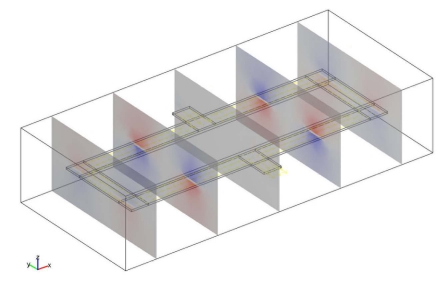
Finite Element Method (FEM) model of a spin-valve based IC sensor.

The FEM can be used in order to model different mechanisms regarding the GMR magnetic field sensors in general and the GMR electric current sensors in particular. For the application of the FEM, a meshing step is always involved. Due to the huge differences in size than can take place between the different elements (nm for the layer thickness, m for the chip dimensions and mm for the width of the current strips), the direct approach to the whole structures is not always possible and partial analysis need to be considered. In Figure 8 some illustrative results on a 2D approach of a spin-valve magnetoresistor with an integrated current strap is shown. Maxwell equations can be directly applied in order to solve the quasi-static magnetic part of the problem (Figure 8(a)). Thermal effects can also be simulated by FEM. With the formalism of heat transfer equations and by applying the well known thermal characteristics of the involved materials, difficult issues such as self heating effects can be quantified (Figure 8(b)). By taking into account higher frequency effects, the application frequency band can be enlarged (Figure 8(c)).

In [58] an illustrative and complete finite element method (FEM) analysis is applied to a particular spin valve based electrical current sensor implementation. In this case, and due to the huge differences in size between the different elements (nm for the layer thickness, *μ*m for the chip dimensions and mm for the width of the current strips), the direct approach to the whole structures was not possible and the analysis was separated into two steps. Firstly, only the PCB was modelled and the 3D distribution of the magnetic field due to the current flow circulating along the PCB track was obtained. Once the magnetic field is known at the positions where the magnetoresistive elements are placed, a linear model of their magnetic dependent resistance is extracted from their linear characteristic. By means of systematic simulations ranging several fabrication parameters (displacements, tilts, …) a fabrication receipt is suggested to avoid long and expensive prototyping steps.

Modelling the micromagnetic response or modelling the acquisition process of semimagnetic semiconductors is also possible [59], but it lies out of the scope of this work.

## 6. Conclusions

″Spaintronics″, or Spanish contribution to the development of GMR based sensors has been demonstrated to be notable. Several research groups are nowadays carrying out fruitful research in GMR primary structures, sensor design and modelling and practical applications. The most of these research lines remain currently active. Applications related to the electric current monitoring are specially important. GMR-based sensors, both commercial and own developed, have been successfully applied applied in different scenarios. Current meters, differential current meters, power meters, IC current and power meters have been developed and tested.

## Figures and Tables

**Figure 5.**
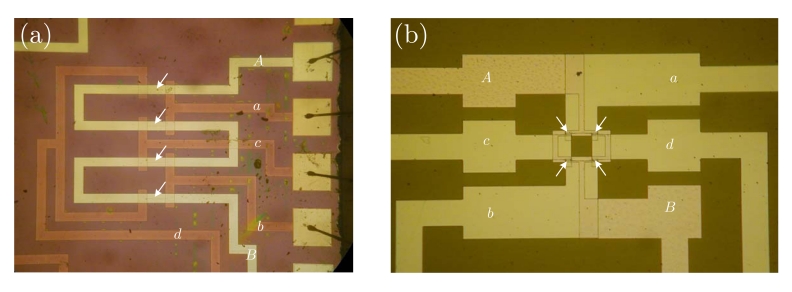
IC current meters. The function scheme and nomenclature can be found in Figure 2. Arrows indicate real magnetoresistor locations. (a) Spin-valve [45], (b) Magnetic tunnel junctions [21].

**Table 1.** Bridge configurations.

Unique element	Half bridge	Full bridge
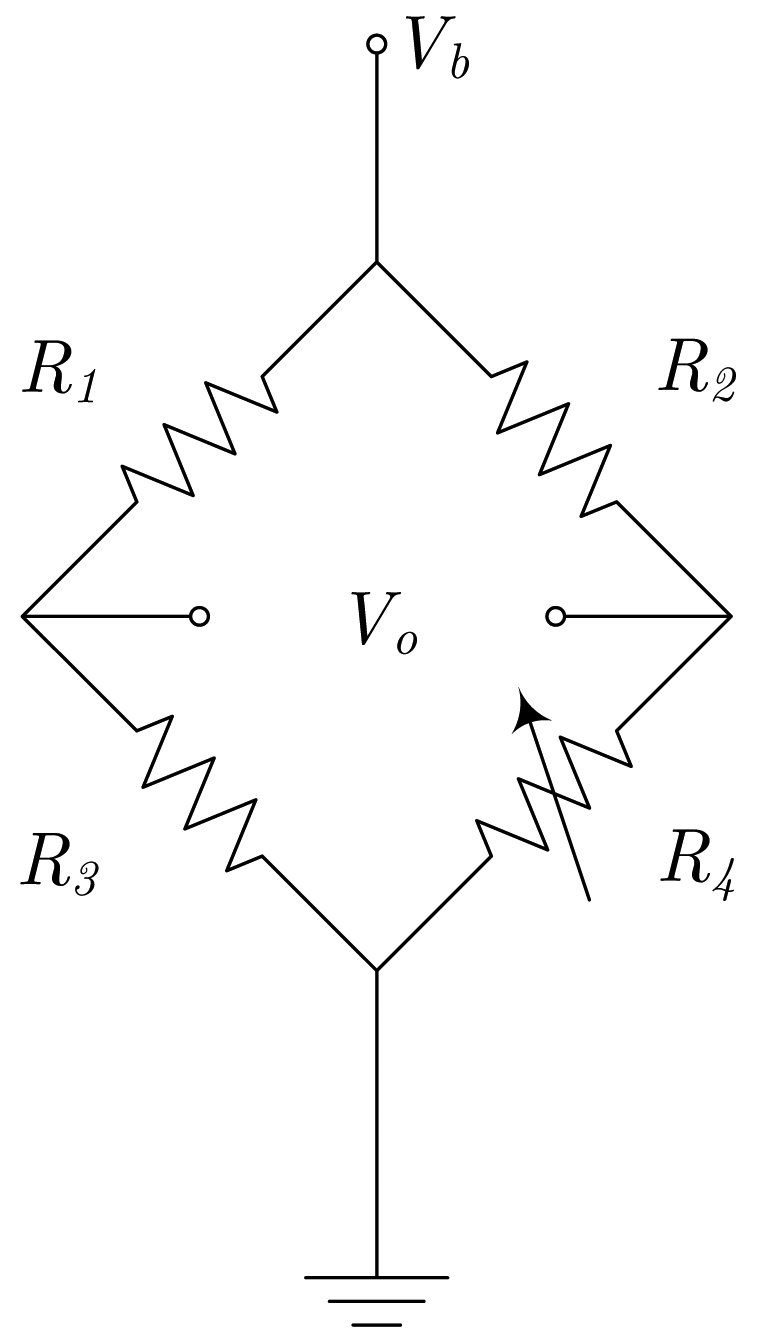	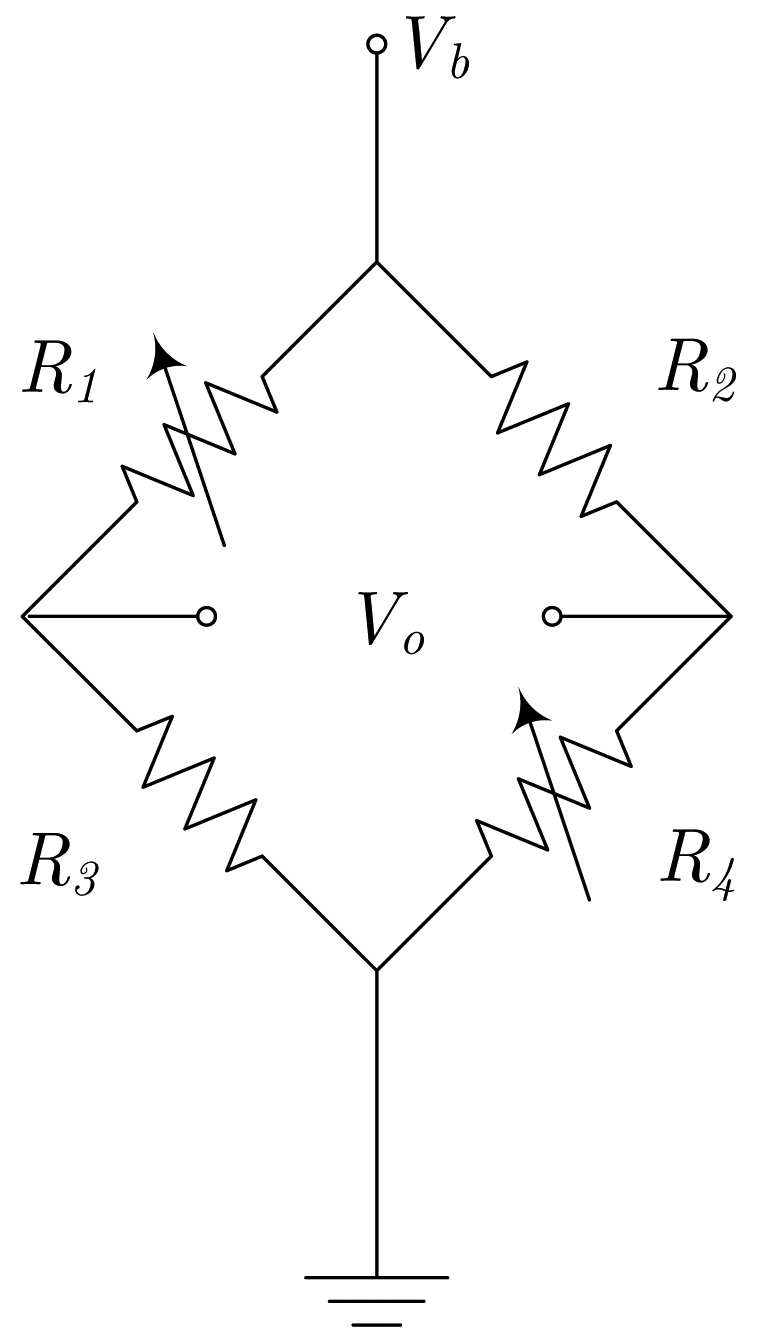	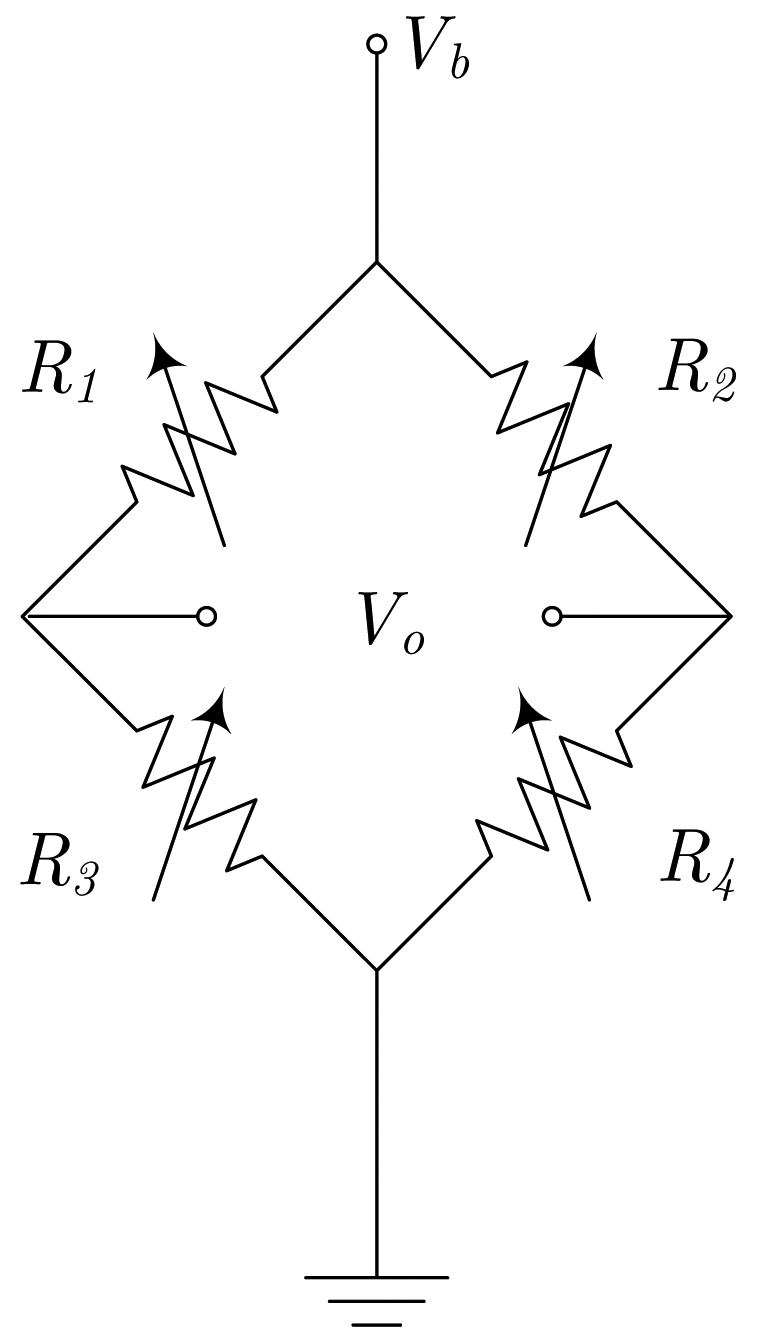
*R*_1_ = *R*_2_ = *R*_3_ = *R; R_4_ = R +* Δ*R*	*R*_2_ = *R*_3_ = *R; R*_1_ = *R_4_ = R +* Δ*R*	*R*_1_ = *R*_4_ = *R+*Δ*R; R_2_ = R_3_ = R* − Δ*R*
$V_{0}=V_{b}\frac{\frac{\Delta R}{R}}{2\left(2+\frac{\Delta R}{R}\right)}$	$V_{0}=V_{b}\frac{\frac{\Delta R}{R}}{2+\frac{\Delta R}{R}}$	$V_{0}=V_{b}\frac{\Delta R}{R}$

**Table 2.** Magnetic field generated by an electric current measurement for different geometries.

∮ **H** dl = I	
cylindrical wire	printed circuit board strap
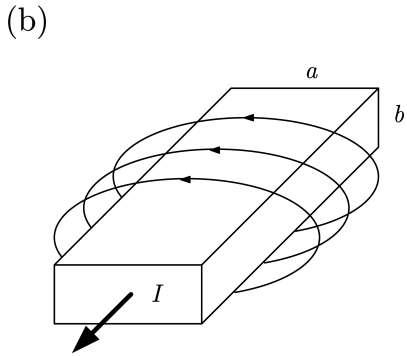	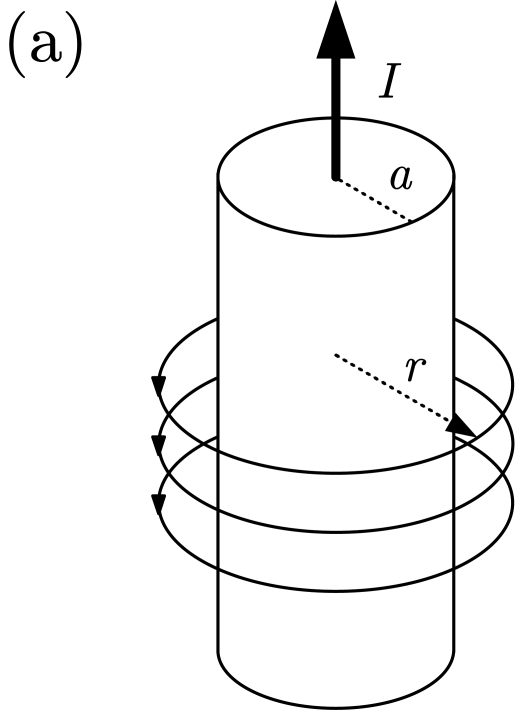
$H(r)=\frac{I}{2\pi r}$	$\text{H}(\text{r})=\frac{1}{4\pi }\int_{s}\begin{array}{l} \frac{\text{J}({\text{r}}^{\prime })\times \text{R}}{{\text{R}}^{2}}\text{d}r^{\prime }\\ \text{R}=r-r^{\prime };R=|R| \end{array}$
